# Twelve Years of Change in Coastal Upwelling along the Central-Northern Coast of Chile: Spatially Heterogeneous Responses to Climatic Variability

**DOI:** 10.1371/journal.pone.0090276

**Published:** 2014-02-28

**Authors:** Guillermo Aravena, Bernardo Broitman, Nils Christian Stenseth

**Affiliations:** 1 Centro de Estudios Avanzados en Zonas Áridas, Facultad Ciencias del Mar, Universidad Católica del Norte, Coquimbo, Chile; 2 Centre for Ecological and Evolutionary Synthesis, Department of Biosciences, University of Oslo, Oslo, Norway; University of Vigo, Spain

## Abstract

We use time-series analyses to characterize the effects of recent climate variability upon the local physical conditions at 11 study sites along the northern-central coast of Chile (29–34°S). Environmental indices show that the 1° Bakun upwelling index in this coastal region has fluctuated in time, starting from a stable period around the 1980's, peaking during the mid 90s, decreasing during the next ten years and increasing at a steep rate since 2010. Upwelling intensity decreased with increasing latitude, showing also a negative correlation with climate patterns (El Niño3 sea surface temperature-SST anomalies and the Multivariate El Niño Index). We hypothesize that the impacts of climate variability on upwelling events seem to be spatially heterogeneous along the region. Non-sheltered locations and, particularly, sites on prominent headlands show an immediate (lag = 0) and negative correlation between local SST, upwelling events and wind stress. We suggest that near-shore thermal conditions are closely coupled to large-scale forcing of upwelling variability and that this influence is modulated through local topographic factors.

## Introduction

The study of the impact of climate variability on regional oceanographic processes is of particular interest in areas of high biological productivity such as the Humboldt Current System (HCS). In the HCS, a productive Eastern Boundary Upwelling Systems (EBUS) providing more than 10% of global fish catch [1], coastal wind-driven upwelling circulation is defined by the interaction of a shallow (50–100 depth) poleward undercurrent along the break of a narrow continental shelf and persistent equatorward surface winds. Wind-driven upwelling brings cold subsurface waters, rich in nutrients and poor in oxygen, to the surface and fuels the large biological productivity characteristic of this coastal zone [2], [3], [4], [5]. Wind-driven upwelling along the EBUS is not a temporally continuous or spatially uniform process, but is often associated to the intensification of equatorward wind events around prominent topographic features [6]. Coastal upwelling along the central-northern coast of Chile is intensified around a few large headlands, such as Punta Lengua de Vaca (30° S) and Punta Curaumilla (33° S), and is driven by south-southwesterly winds events during austral spring and summer [7], [8], [9]. Coastal upwelling patterns can be modified by equatorial processes such as warm El Niño-Southern Oscillation events (ENSO), which lead to increased sea level pressure (SLP) and sea surface temperature (SST) in the eastern Pacific and a decrease (or reverse) of coastal equatorward winds and primary productivity [3], [4]. Similarly, the poleward propagation of equatorial Kelvin waves along the coast of South America can modify coastal upwelling circulation in north-central Chile through changes in the depth of the thermocline and wind patterns [10], [11], [12], [13].

Recent trends in the intensity and magnitude of the upwelling circulation have been detected in several EBUS that seem consistent with the Bakun hypothesis [2],[14], [15], [16], [17], [18], [19]. Although broad effects of climate trends for the HCS have been modelled (e.g. [20], [21], [22]), scarce information is available to date of the local effects of large-scale climate variability in the HCS.

Increased intensity of upwelling has been implicated in the observed changes in the physical and biogeochemical conditions of the California Current System (CCS) [23], [24] and the decrease of nitrogen in the Benguela Current System [14]. It has also been reported that recent changes in the frequency (less), magnitude (stronger) and duration (longer) of upwelling events in the CCS is changing local oceanographic conditions and the structure and functioning of rocky shore communities [25], [26].

In the case of the southeastern Pacific, while a general ocean warming trend has been observed over the past decade as a consequence of the fact that recent ENSO events have been of the central Pacific or Modoki variant [27], with maximum surface temperature anomalies located in the central rather than eastern Pacific [28], [29]. Recent studies have also detected a near-shore cooling related to an intensification of the coastal southerlies over the past decades [19], [30]. Coupling results from model ensembles and observations, decreased temperature at coastal stations along the coast of central Chile have been related to a Niña-like intensification of the South Pacific Anticyclone [19], [20]. This climate pattern has strengthened the alongshore pressure gradient and increased coastal upwelling and offshore transport, due to Ekman transport and Ekman pumping, respectively [9], [20].

Lack of high-resolution and long-term local observations of the coastal ocean have hindered a quantitative evaluation of local oceanographic responses in the Chilean coast to recent climate trends (e.g. [31], but see [19]). Here, we employ time-series analyses using equatorial climate indices, the Bakun upwelling index and satellite offshore temperatures to examine the effects of climate variability on local, long-term near-shore SST records along the northern-central coast of Chile. As the spatial distribution of upwelling is modified by coastal topography, we hypothesize that the effects of large-scale climatic variability on coastal upwelling are locally heterogeneous along the latitudinal gradient considered.

## Methodology

### Ethics Statement

This study was conducted as part of the activities carried out by both the ECIM (Estación Costera de Investigaciones Marinas at Pontificia Universidad Católica de Chile) and the Changolab (at the Centro de Estudios Avanzados en Zonas Áridas, CEAZA) group, and approved by their respective bioethics committees. No specific permits were required for the described fieldwork, because they were based on water temperature records collected at all locations, which were not privately-owned. Non-destructive manipulation of endangered or protected species was required.

### Study area

**Figure 1:**
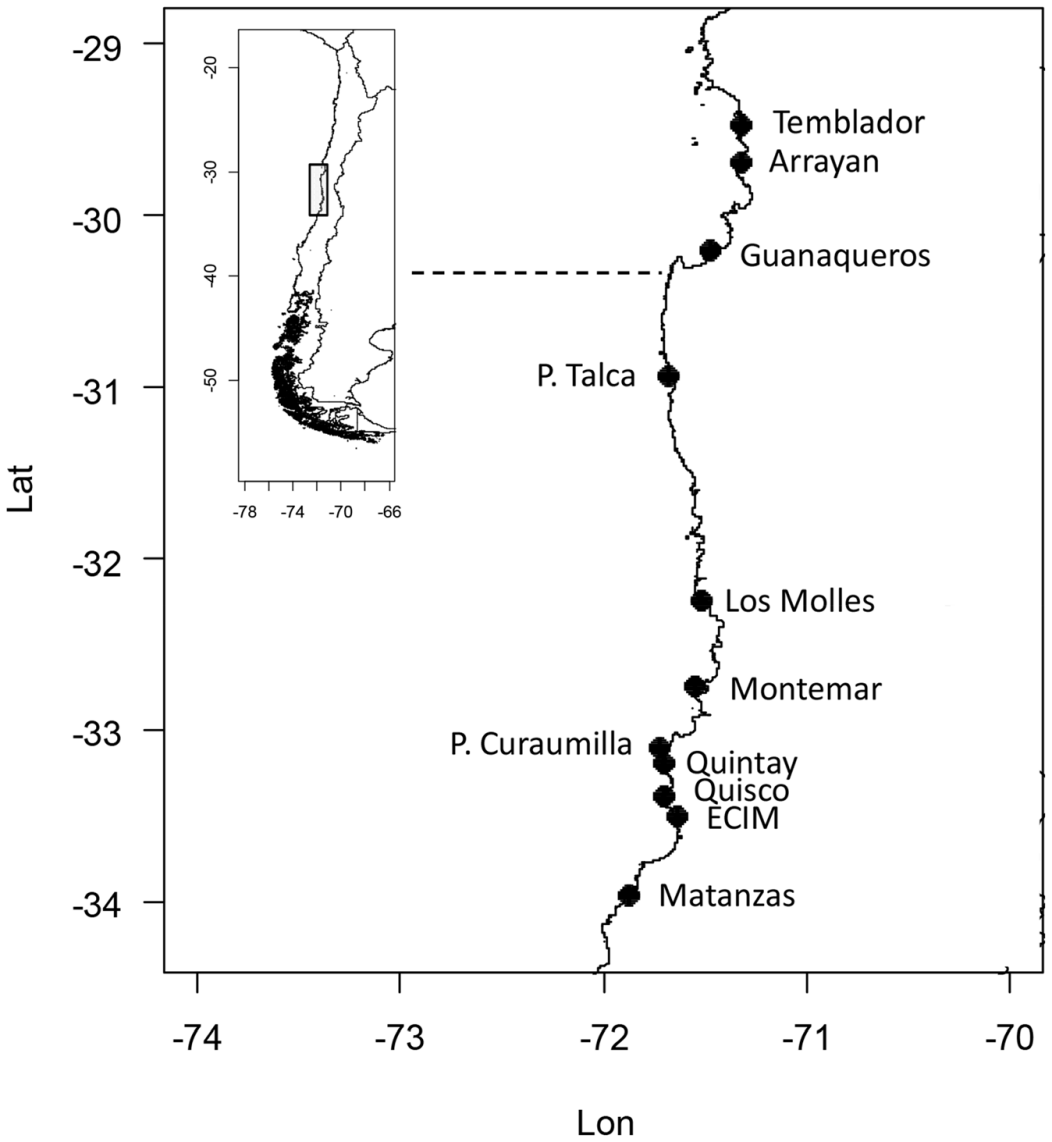
Study Area. Sampling locations showing coastline exposure division (dotted line) between a more protected (from Temblador to Arrayan) and a more exposed coastline (from P. Talca to Matanzas), and upwelling locations (P. Talca, P. Curaumilla and Quintay) as described in the text.

We used 12 years (1999–2010) of monthly mean SST data collected at 11 sites located along the northern-central cost of Chile (29°–34°S, Figure 1). The study area is characterized by a zone with a more protected coastline from 29–30° S and an area with a more exposed coastline from 30 to 34° S that include some known upwelling centers (at 30 and 33° S, Figure 1) [32], [33], [34] and small embayments.

### Climate indices and oceanographic data

To characterise ENSO variability we used the Multivariate El Niño Index (MEI), which combines sea-level pressure, zonal and meridional components of the surface wind, sea surface temperature, surface air temperature, and total cloudiness fraction of the sky for the tropical Pacific using the first unrotated Principal Component of all six observed fields combined [35] (http://www.esrl.noaa.gov/psd/enso/mei/). We also used the monthly Bakun upwelling index (UI) and the north-south component of wind stress (N/m^2^) in all the coastal latitudinal bins (29° to 34°S) of our study locations to examine upwelling intensity at local scale (see Figure 1). PFEL calculates coastal upwelling indices from 1°-resolution sea level pressure fields obtained from the US Navy Fleet Numerical Meteorology and Oceanography Center. The index is based on estimates of Ekman mass transport of surface water due to wind stress and the Coriolis force. Positive values, the result of equatorward wind stress, are an estimate of the amount of water upwelled from the base of the Ekman layer (m^3^·s^−1^·100 m^−1^ of coastline). Negative values imply downwelling, accompanied by the onshore advection of surface waters. For a detailed description, please see http://www.pfeg.noaa.gov/products/las/docs/wind_from_pressure.nc.html). Additionally, and to examine the relations between coastal SST in central-northern Chile and equatorial variability we examined monthly SST mean Nino3 index (EN3SST) which is given by the mean anomalous SST from the region bounded by 90°W–150°W and 5°S–5°N.

### Sea surface temperatures

To quantify oceanographic conditions adjacent to the study sites we used monthly mean SST from the Advanced Very-High Resolution Radiometer (AVHRR) processed with the Pathfinder version 5 algorithm [36]. We used level-3 data available as weekly composites at a nominal resolution of 4 km (http://www.nodc.noaa.gov/sog/pathfinder4km/). The off-shore temperature adjacent to each site was determined by averaging all cloud free pixels within the 24 km×24 km bin centered on the site. The use of a relatively large bin size was necessary as missing pixels were common in the near-shore [37]. Local validations of SST estimations using this method presented in [38] for several sites in central California (US) suggest that monthly means over the 20×24 km area describe appropriately oceanographic variability at the site scale (see also [39] for the central coast of Chile).

Finally, we characterized the local near-shore oceanographic conditions using SST derived from submersible temperature data loggers (Tidbit StowAway; ONSET, Pocasset, Massachusetts, USA) anchored at between 1.0 and 1.5 m below sea level at low tide (see also [40], [41]). Temperature loggers are self-contained and disposable, temperature records are retrieved on a montlhy or bi-monthly basis and loggers are frequently exchanged for new instruments to avoid drift. In-situ calibration has been regularly conducted by deploying pairs of instruments on the same site.

### Statistical analysis

#### Spatio-temporal trends

Monthly interpolated raw data and anomalies of time-series were visually examined to assess their inter-annual evolution and to inspect differences in the fluctuation patterns. To statistically check for differences between satellite and temperature data loggers, we also used seasonal plots and paired t-tests to compare means derived from cold (winter: Jun–Sep) and warm seasons (summer: Dec–Mar).

#### Relationships between variables

In order to document low frequency upwelling patterns, we used wavelet spectrum (WS) and wavelet coherence (WCO) analysis. Wavelet analyses allow non-stationarity in noisy time series by performing a localized spectral decomposition of the signal, determining the dominant modes of variability in time and scale [42], [43]. The wavelet methodology is described in detail in Methods S1.

To infer cause-effect relationships between time-series over time, prewhitening cross-correlation functions (CCFs) were fitted as described by [44]. Prewhitened CCFs allow to cope with non-stationarity and dependence of the data, to eliminate any spurious correlation between series and to reveal underlying relationships (see for example [44], [45]). We first used a seasonal Autoregressive Integrated Moving Average model (SARIMA) on the input (*X*) series and then filtered the output series (*Y*) with an identical SARIMA model to fit the CCFs between both filtered series [44], [45], [46]. The regression model between both (X,Y) series may in its general form be expressed as: 
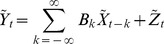
where Y is the prewhitened output (*Y*) variable at time *t*, X the prewhitened input (*X*) variable at time *t* that is, at the same time, independent of Z, which may follow any SARIMA (*p,d,q*) (*P,D,Q*) model (estimated initially for *X*), and *B* is the autoregressive coefficient to be estimated at each lag *k*. For more detailed information, please see [44], [45]. All analyses were performed using the R package 2.14 [47].

## Results

### Spatio-temporal variability

**Figure 2:**
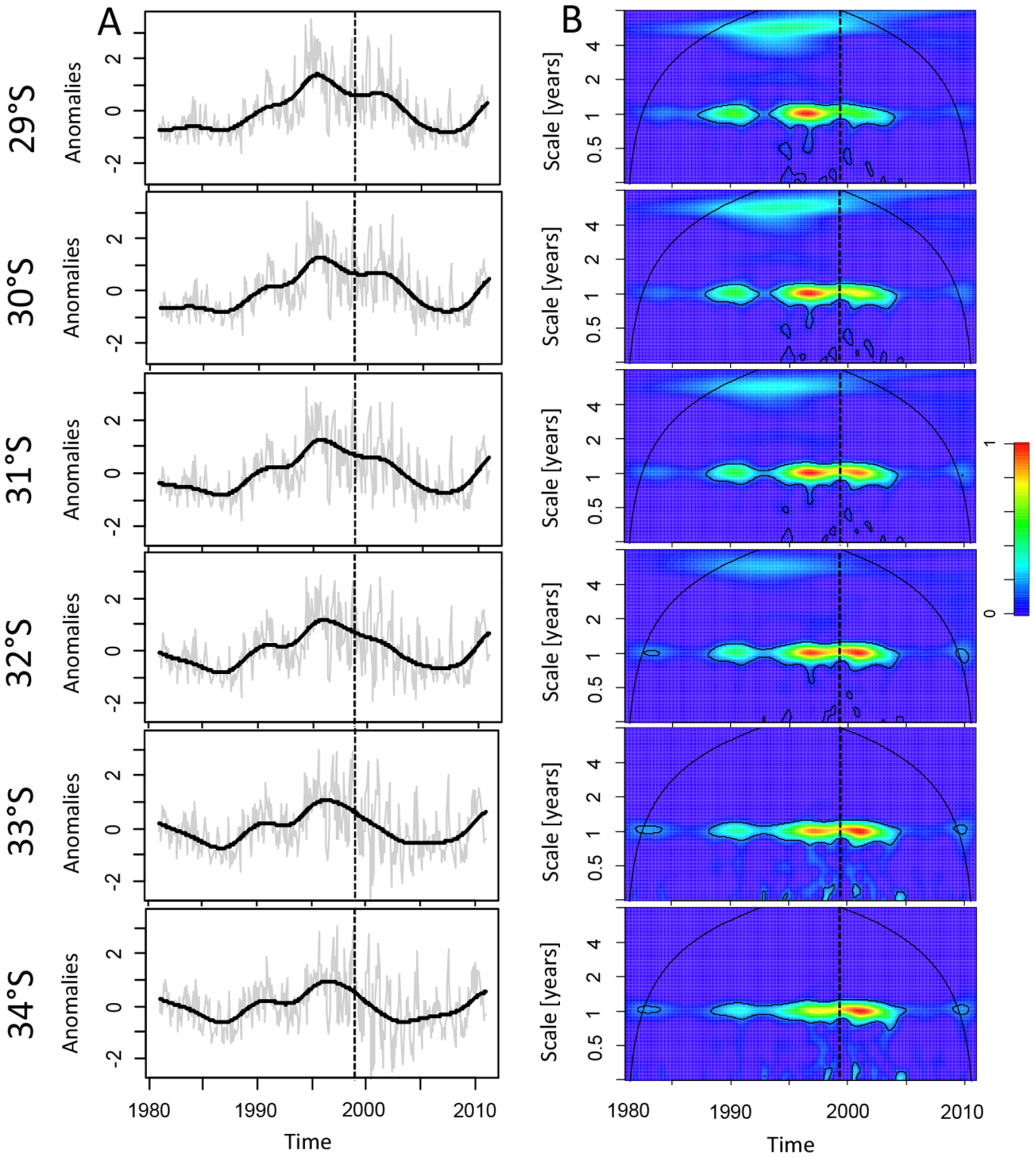
Trends of time series upwelling (UI). (A) UI anomalies time series (in grey) and trends using splines (black line) and (B) wavelet power spectrum (WPS) from 1980 to 2010 (including our time period span 1999–2010, dotted line). For the WSP, the area marked by the black lines indicates the cone of influence where edge effects become important (>95% confidence).

**Figure 3:**
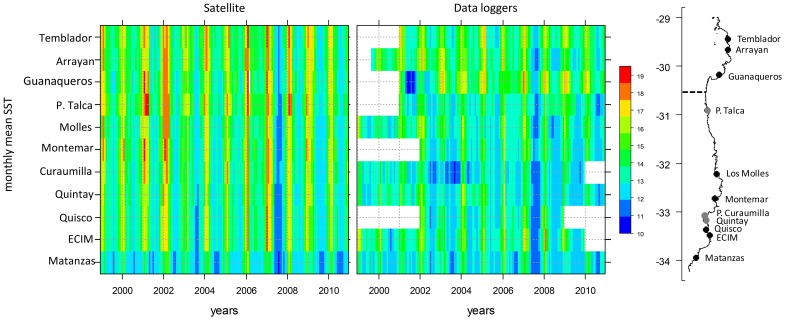
Spatio-temporal trends of sea surface temperature (SST). Monthly mean SST time-series derived from satellite imagery and data loggers. The spatio-temporal trend in satellite evidences a clear seasonal patterns with cold (blue) and warm (red) periods in contrast with data loggers with predominance cold conditions (blue) in the exposed coastline (from P. Talca to Matanazas, dotted line). White spaces correspond to missing values.

Decadal variability in UI was evident over the record examined (Figure 2). While UI varied little during the 1980's, upwelling transport increased until the middle of 90's, then decreased until the mid 2000's before increasing again until the end of 2010. Satellite SST data revealed anomalous warm (annual mean temperature >18°C) years during 2001–02 and 2006–07, and an extremely cold (annual mean temperature <12°C) year during 2007. In contrast, water temperatures derived from loggers show markedly weaker seasonal pattern, and a tendency for colder near-shore conditions from P. Talca south to Matanzas (∼30.5 to 34°S, Figure 3 in blue) relative to off-shore. While there were no strong El Nino events during our study period, the anomalous cold off- and near-shore conditions during 2007 (Figure 3) could correspond to the occurrence of La Niña. Both satellite and loggers also revealed a common response of SST records to this cold conditions, showing that those locations with a straight coastline and, particularly, at points (as those located southward in our study area) seem be more susceptible to the impact of climate patterns (Figure 3). Significant differences exist between minimum and maximum values derived from satellite data and temperature data loggers. Satellite derived-SST were warmer than those recorded by data loggers, which revealed colder temperatures at the shore. While the satellite SST record possesses a smooth meridional gradient, with higher temperature (maximum: 18.82°C, minimum: 11.62°C, mean: 14.79°C) in the northern stations (from Temblador to Guanaqueros, 29 to 30°S) and minimum values southward (Matanzas, maxima: 18.10°C, minima: 11.30°C, mean: 14.08°C), near-shore SST is found to depend more strongly on the local coastal geometry, and in fact the lowest temperatures were observed at intermediate locations such as P. Curaumilla (maxima: 10.°C, minima: 11.30°C, mean: 14.08°C). From the subset of SST records derived from loggers (Figure 3), it is apparent that sites located on headlands (i.e. P. Curaumilla) were always colder than adjacent sites. The cross-shore SST gradient was found to be more intense during summer seasons and at locations characterized as upwelling centres (P. Talca, P. Curaumilla and Quisco) (Figure S1). Differences between satellite and logger SST during summers (Table 1) were particularly large (with t-values >7.5 and p<0.001) across sites from ∼30.5° to 34°S, whereas differences in winters were mostly significant (with t-values >4.1 and p<0.001) from ∼30.5° to 34°S (except at ECIM). The paired t-test also revealed marked differences at P. Talca (summers: t-values  = 6.88, p-value <0.001, and winters: t-values  = 17.78, p-value <0.001), P. Curaumilla (summers: t-values  = 7.94, p-value <0.001, and winters: t-values  = 22.68 and p-value <0.001) and Quintay (summers: t-values  = 4.12, p-value <0.001, and winters: t-values  = 19.14, p-value <0.001).

**Table 1:** T-paired test showing significant differences between satellite and loggers-derived SST records during winter (Jun–Sep) and summer (Dec–Mar).

	summer			winter		
site	cross-shore differences °C	t-value	p-value	cross-shore differences °C	t-value	p-value
Temblador	1.299	6.827	<0.001	0.453	4.385	<0.001
Arrayan	1.103	6.515	<0.001	0.317	3.981	<0.001
Guanaqueros	1.340	3.694	0.001	0.421	2.307	0.029
P. Talca	3.053	17.777	<0.001	0.738	6.885	<0.001
Los Molles	2.256	11.812	<0.001	0.506	6.849	<0.001
Montemar	2.415	27.783	<0.001	0.556	5.078	<0.001
P. Curaumilla	3.066	22.683	<0.001	0.678	7.938	<0.001
Quintay	2.614	19.139	<0.001	0.436	4.120	<0.001
Quisco	2.068	17.681	<0.001	0.512	8.693	<0.001
ECIM	0.939	7.505	<0.001	0.077	1.040	0.306
Matanzas	2.304	18.112	<0.001	0.530	5.823	<0.001

### Relations between variables

Wavelet coherence and phase between UI along the coast of central-northern Chile and EN3SST are shown in Figure S2. Wavelet coherence indicates that, as expected, UI was negatively related to equatorial processes over annual-scales, but also that the relationship is latitude-dependent being more permanent southward. The significant coherence over annual scales was intermittent in the northern sector, possibly through the more seasonal structure of upwelling towards the southern region [48].

**Figure 4:**
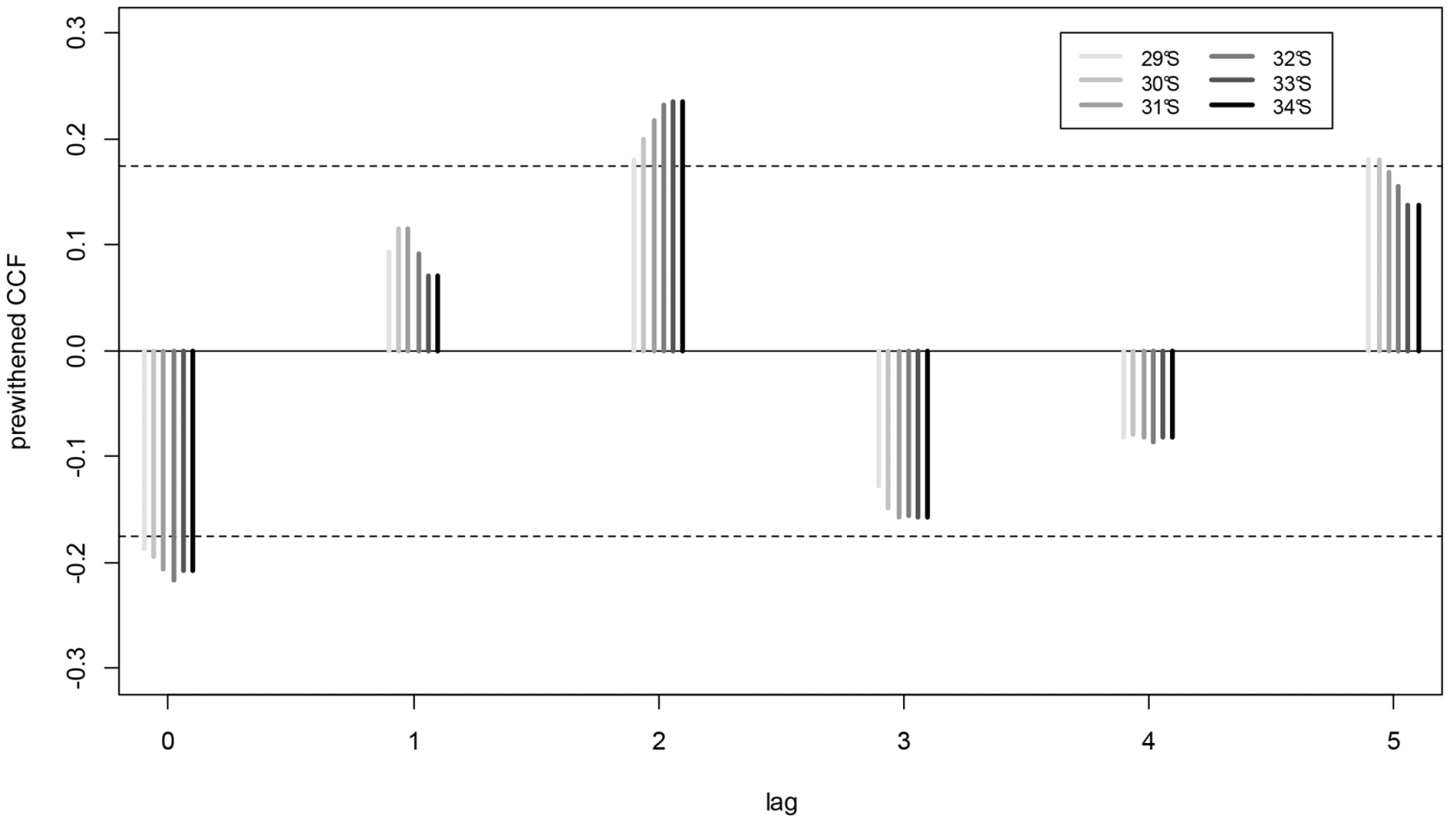
Cross-correlations between MEI and UI. Prewhitened cross-correlation functions (CCFs) between monthly time-series of the Multivariate EL Niño Index (MEI) and the Upwelling Index (UI) estimated for different latitude: 29° (ccf: −0.187; t: −2.151; p-value: 0.033), 30° (ccf: −0.194; t: −2.242; p-value: 0.003), 31° (ccf: −0.206; t: −2.378; p-value: 0.019), 32° (ccf: −0.216; t: −2.499; p-value: 0.014), 33° (ccf: −0.208; t: −2.402; p-value: 0.018) and 34°S (ccf: −0.182; t: −2.096; p-value: 0.038). Standard error limits are shown as dotted lines. The significant and inverse correlations are mostly observed at Lag = 0.

**Figure 5:**
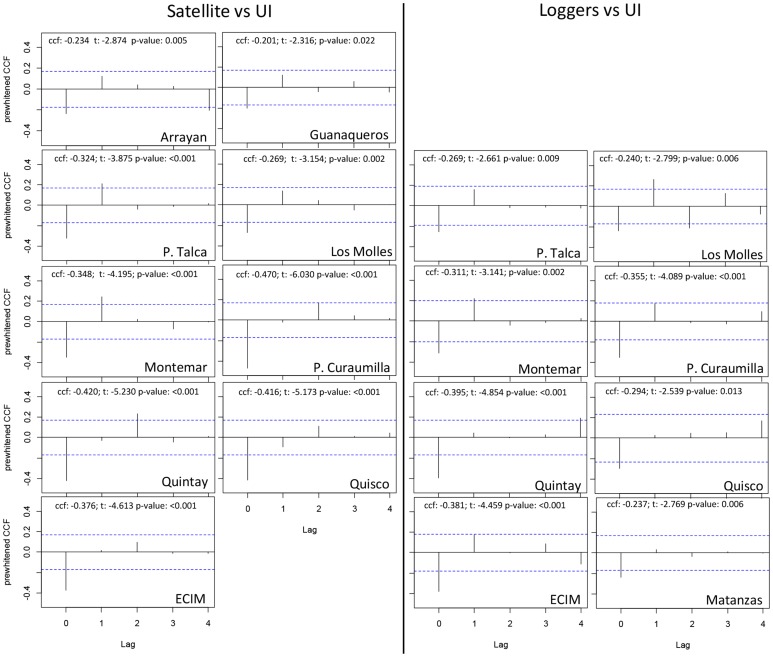
Cross-correlations between SST and UI. Prewhitened cross-correlation functions (CCF) between monthly off- (satellite derived-SST) and near-shore (loggers-derived SST), and UI at all regular monitoring sites. Standard error limits are shown as dotted lines. The significant and inverse correlations are mostly observed at Lag = 0.

**Figure 6:**
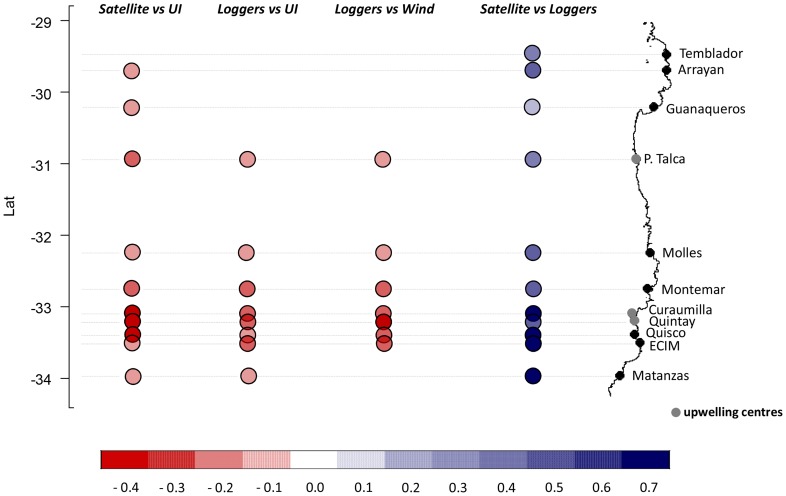
Cross-correlations between variables. Summary of CCF (correlations values) obtained between the different physical variables (SST, upwelling index-UI, and wind-stress) considered along the northern-central Chilean coast. More significant CCFs were obtained in the more exposed coastline (from P. Talca to Matanzas) with highly significant values mainly observed towards southern locations.

Prewhitened CCFs confirmed the negative and immediate (lag = 0) connection between MEI and UI scales across the range of latitudes examined (29° to 34° S). Lag-0 correlations were more strong southwards (Figure 4). Monthly UI were negatively and immediately (lag-0) correlated with SST time-series derived from AVHRR and loggers (Figure 5). Negative lag-0 correlations between off-shore SST and UI were observed along the whole area area whereas correlations between near-shore SST and UI were mostly observed in the exposed coastline from P. Talca to Matanzas (Figure 5). Figure 6 summarizes the CCFs relationships obtained between SST and both upwelling and wind-stress, correlations that were mainly obtained in the more exposed coastline (from P. Talca to Matanzas) with highly significant p-values mainly observed towards southern locations.We find that wind-stress was negatively correlated with water temperature from data loggers at lag-0 at locations on the exposed coastline (Figure 6). As expected, we observed that surface temperatures derived from satellite and data loggers were positively and immediately (lag = 0) correlated along the study area (Figure 6).

## Discussion

At global scale, several studies have shown that the spatial and temporal characteristics of upwelling can change following global climate trends, in broad agreement with Bakun's hypothesis [2], [16], [17], [18] [25]. Changes in the frequency, magnitude and duration of upwelling events could have a large impact on the coastal marine ecosystem. Along the Chilean coast, two zones can be identified according to the alongshore winds seasonality: a northern region (18°–30°S) characterized by its year–round favorable upwelling winds [7]; and a central region (30°–41°S) characterized by its strong upwelling seasonality (during the austral spring and summer) that is especially marked in its southern part (e.g., [49]). Therefore, the predominance of seasonal variability in our central Chilean sites is not surprising (Figure 2). Moreover, upwelling fluctuated in time and space (Figure 2), showing that coastal upwelling, and in general nearshore SST shows a strong low-frequency variability signal at mid latitudes [50] along the HCS. Therefore, we suggest that changes in upwelling patterns seems to be more complex than a direct response to global climate trends and that local conditions, in particular coastline topography, can strongly modify the impact of large-scale processes. Studies show that the hypothesis suggested by Bakun is not satisfactorily corroborated at local scales possibly because the scale of the coastal intensification may be small relative to the scales that are appropriately reflected in the standard models [51]. These authors have proposed that other variables such as water vapour (here not considered) could be playing a key role in the complex climate-ocean interaction [51].

Our results derived from coherence and phase analysis (and corroborated with CCFs) revealed a negative and significant correlation between UI and EN3SST (and between UI and MEI with CCFs), which were more persistent southward (Figure S2). Changes in equatorward-favorable upwelling winds in northern Chile (north of 25°S) do not seem to be clearly forced by ENSO [50] in contrast with higher latitudes where equatorial variability may impact local upwelling strength (e.g. [52]). Changes in intensity and frequency of upwelling as a latitude-dependent response to climate change have also been reported for others EBUS such as the California Current [16], [53]. Recent modelling studies indicate an increase of the equatorward alongshore winds on the Chilean coast under a future altered climate scenario [20], [54]. Accordingly, we suggest that upwelling-favorable winds are modulated, as a function of the latitude, by climate patterns. Nevertheless, we emphasize the need to expand our study towards austral (higher) latitudes along the HCS in order to test such hypothesis.

CCFs showed that satellite SST was negatively correlated with upwelling events between Arrayan and ECIM whereas data loggers show negative relationships with upwelling only at sites located on the more exposed coastline (from P. Talca to Matanzas). In addition, we observed that seasonal patterns in near-shore surface temperatures (derived from loggers) were not consistent with those derived from satellite suggesting that the AVHRR platform is not completely able to capture higher frequency variability at local scale [55], [56]. Such differences, mostly observed during summers are indicative of a thermal bias in near-shore waters. A warm near-shore bias (>4°C from January to March) derived from the Pathfinder climatology is explained as a consequence of the strong cross-shore SST gradients in this upwelling zone, which cannot be satisfactorily represented by satellite imagery [57]. Although satellite products have been successfully used for monitoring inner-shelf waters, any high resolution SST (i.e. a radius of several km) for a specific coastal location is often precluded by cloud cover and/or poor data quality (i.e. the quality of model selected and/or smoothing fitted) [34], [57], a handicap that may be exacerbated in this changing upwelling ecosystem. These near- and off-shore thermal differences could be also explained due to the fact that *in situ* (derived from loggers) surface temperature patterns are linked to topography [34], [58], [59]. In particular, sites characterized by headlands and points can intensify coastal upwelling and serve as the source location for the generation of eddies and filaments that transport cold surface waters off-shore [33], [41], [60]. In fact, we observed higher differences at locations with a more exposed coastline and sites topographically characterized as points, where strong coastal upwelling has been reported [32], [60]. These results are also consistent with those reported by [34], who found high values of a multivariate upwelling index (derived from *in situ* surface temperatures) in these three exposed locations (P. Talca, P. Curaumilla and Quintay). In contrast, sites along the more protected coastline (from Temblador to Guanaqueros) may be separated from the recently upwelled waters by frontal structures. This is a classic pattern of circulation associated to bays [6], [61] has been reported by Lagos *et al*. [41] for the northern coast of Chile. This reduced off-shore Ekman transport was already noted by [34] for protected coastline sites (from Temblador to Guanaqueros). Thus, topography seems to be an important factor in explaining the variability of *in situ* thermal conditions and the magnitude of upwelling along the central-northern Chilean coast [33], [34], [62].

Along the northern-central coast of Chile, we observed a chain of effects from climate patterns to coastal upwelling that, in turn, is influencing local oceanographic conditions. Our results provide evidence that large-scale climate leads to changes in upwelling patterns. We also suggest that nearshore surface temperatures are being mostly influenced by prolonged cold events associated to the impact of upwelling episodes, which tend to be persistent at zones with more exposed coastline and, in particular, at locations on or around prominent topographic features. A major ecological implication of our study is that such changes can impact the dynamics and structure of coastal resources [51], [25]. In this way, our results increase our understanding about the physical links between climate and local oceanographic variability along the HCS. We suggest that further studies should incorporate additional local measurements to improve our understanding on the dynamics of these productive coastal ecosystems. The lack of a higher temporal resolution (e.g. at daily scale) in the upwelling data analyzed here implies that our results are limited to monthly scales. Incorporating higher resolution long-term records may shed further light on the dynamics of this large ecosystem.

## Supporting Information

Methods S1 **Detailed description of the methodology and software used for performing wavelet coherence and phase.** A brief detail about the calculation for differencing time series is also described.
(DOC)

Figure S1 **Seasonal trends of differencing (▽Y_t_ = Y_t_−Y_t-_1) SST time series for satellite (in black) and data logger (in grey) at different sampling locations.** Lines represent the means of differencing values for each month.
(TIF)

Figure S2 **Wavelet coherence and phase between the upwelling index and sea surface temperatures-Niño, computed with data from 1981 to 2010 (included our study time span: 1999–2010, dotted line).** The area marked by the black lines indicates the cone of influence where edge effects become important. The solid black contour encloses regions of >95% confidence.
(TIF)
