# Supplementary material for: Twelve Years of Change in Coastal Upwelling along the Central-Northern Coast of Chile: Spatially Heterogeneous Responses to Climatic Variability
Source: PLoS One. 2014 Feb 28;9(2):e90276. doi: 10.1371/journal.pone.0090276 (PMC3938675; doi:10.1371/journal.pone.0090276)
Supplement: Methods S1 — Detailed description of the methodology and software used for performing wavelet coherence and phase. A brief detail about the calculation for differencing time series is also described. (DOC) [file pone.0090276.s001.doc]

**Supporting Information Methods S1**

Twelve years of change in coastal upwelling along the central-northern coast of Chile: spatially heterogeneous responses to climatic variability

Guillermo Aravena, Bernardo Broitman, Nils Christian Stenseth

***Wavelets Analysis***

To compute wavelet spectrum (WS) and wavelet coherence (WCO) we used the methodology described by [1],[2], and as performed in the R package SOWAS. The SOWAS package also uses the Morlet wavelet, which provides a good balance between scale (frequencies) and time localizations [3],[4], and is one of the more appropriated mother functions in terms of reproducing the frequency decomposition of the signal [5]. It has often been successfully used in the study of environmental [6],[7],[2] and ecological variables [8],[9],[10],[11],[12].

***Wavelet power spectrum***

Transferring a concept of Fourier analysis, a wavelet power spectrum (WPS) can be defined as the wavelet transformation of the autocorrelation function. Following the Wiener-Khinchin theorem, this can be implemented as follows:

$$WPS_{i}(s)=\left\langle W_{i}(s)W_{i}(s)*\right\rangle$$

where the brackets denote expectation values.
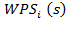
 describes the power of the signal *x(t)* at a certain time *ti* on a scale *s*.

***Wavelet Coherence***

The WCO is defined as the amplitude of the wavelet cross spectrum (WCS) normalized by the wavelet spectrum of each signal [1],[13], that is:

where
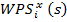
 (s) and
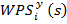
 are the wavelet power spectrum of the time series, *x(ti)* and *y(ti)*, respectively, and 〈〉 denotes a smoothing operator, which is performed by means of the convolution with a constant-length window function both in the time and scale [1], [2], [13].

The phase coherence is defined as reported by [13]:

$$J_{i}(s)=\tan^{-1}\frac{lm\left(\left\langle WCS_{i}(s)\right\rangle\right)}{Re\left(\left\langle WCS_{i}(s)\right\rangle\right)}$$

where
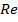
 () and
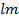
 () mean the real and imaginary parts of the
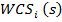
, respectively. The phase coherence provides information about the possible delay in the relationship (e.g. the time series are either in or out of phase) [13]. Further methodological details of phase coherence are given in [1] [13].

***Differencing time-series***

In order to consider the non-stationary condition of SST, we calculated the differences of successive Y-values, denoted ▽Y_t_ = Y_t_ - Y_t-1_. Clearly, many real time series cannot be reasonably modeled by stationary processes since they are not in statistical equilibrium but are evolving over time. However, we can frequently transform nonstationary series into stationary series by simple techniques such as differencing [14].

**References**

1. Maraun D, Kurths J (2004) Cross wavelet analysis. Signiﬁcance testing and pitfalls. Nonlinear Process Geophys 11: 505-514.

2. Maraun D, Kurths J, Holschneider M (2007) Nonstationary Gaussian processes in wavelet domain: synthesis, estimation and signiﬁcance testing. Phys Rev E 75: 016707.

3. Grinsted A, Moore JC, Jevrejeva S (2004) Application of the cross wavelet transform and wavelet coherence to geophysical time series. Nonlinear Process Geophys 11: 561-566.

4. Mi X, Ren H, Ouyang Z, Wei W, Ma K (2005) The use of the Mexican Hat and the Morlet wavelets for detection of ecological patterns. Plant Ecol 179: 1-19.

5. Kirby JF (2005) Which wavelet best reproduces the Fourier power spectrum?. Comput Geosci 31: 846-864.

6. Torrence C, Compo GP (1998) A practical guide to wavelet analysis. B Am Meteorol Soc 79: 61-78.

7. Torrence C, Webster PJ (1999) Interdecadal changes in the ENSO-Monsoon system. J Clim 12: 2679-2690.

8. Mènard F, Marsac F, Bellier E, Cazelles B (2007) Climatic oscillations and tuna catch rates in the Indian Ocean: a wavelet approach to time series analysis. Fish Oceanogr 16: 95-104.

9. Corbineau A, Rouyer T, Cazelles B, Fromentin JM, Fonteneau A, Mènard F (2008) Time series analysis of tuna and swordﬁsh catches and climate variability in the Indian Ocean (1968–2003). Aquat Living Resour 21: 277-285.

10. Rouyer T, Fromentin JM, Stenseth NC, Cazelles B (2008) Analysing multiple time series and extending signiﬁcance testing in wavelet analysis. Mar Ecol Progr Ser 359: 11-23.

11. Hsieh CH, Chen CS, Chiu TS, Lee KT, Shieh FJ, Pan JY, Lee MA (2009) Time series analyses reveal transient relationships between abundance of larval anchovy and environmental variables in the coastal waters southwest of Taiwan. Fish Oceanogr 18: 102-117.

12. Polanco J, Ganzedo U, Sáenz J Caballero-Alfonso AM, Castro-Hernández JJ (2011) Wavelet analysis of correlation among Canary Islands octopus captures per unit effort, sea-surface temperatures and the North Atlantic Oscillation. Fish Res 107: 177-183

13. Cazelles B, Chavez M, Berteaux D, Mènard F, Vik JO, Jenouvrier S, Stenseth NC (2008) Wavelet analysis of ecological time series. Oecologia 156: 287-304.

14. Cryer JD, Chang K-S (2008) Time Series Analysis: With Applications in R. Second Edition. Springer. 491 pp.
