# Supplementary figures and images for: Twelve Years of Change in Coastal Upwelling along the Central-Northern Coast of Chile: Spatially Heterogeneous Responses to Climatic Variability
Source: PLoS One. 2014 Feb 28;9(2):e90276. doi: 10.1371/journal.pone.0090276 (PMC3938675; doi:10.1371/journal.pone.0090276)

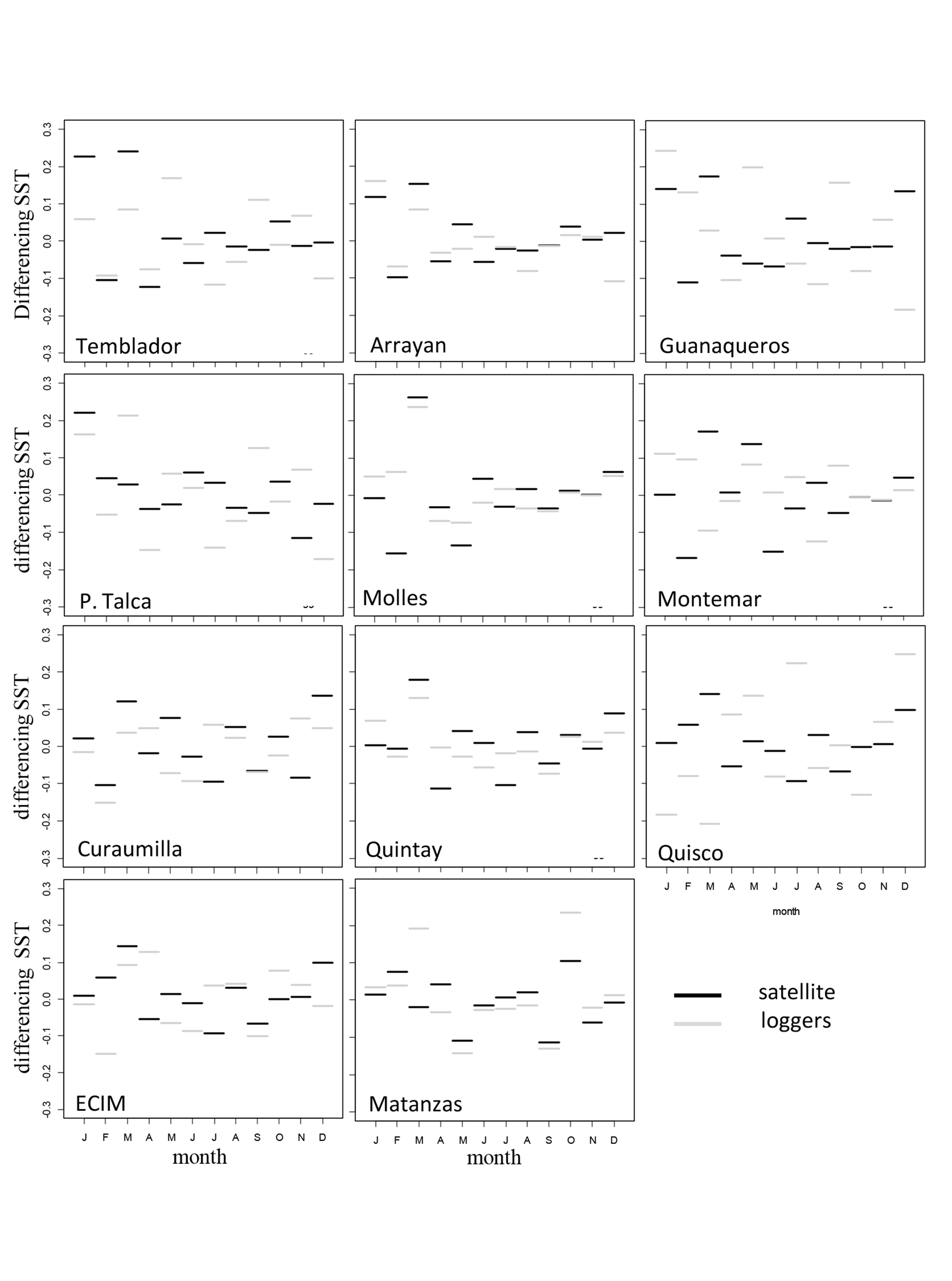

Supplement: Figure S1 — Seasonal trends of differencing (▽Yt = Yt−Yt-1) SST time series for satellite (in black) and data logger (in grey) at different sampling locations. Lines represent the means of differencing values for each month. (TIF) [file pone.0090276.s002.tif]

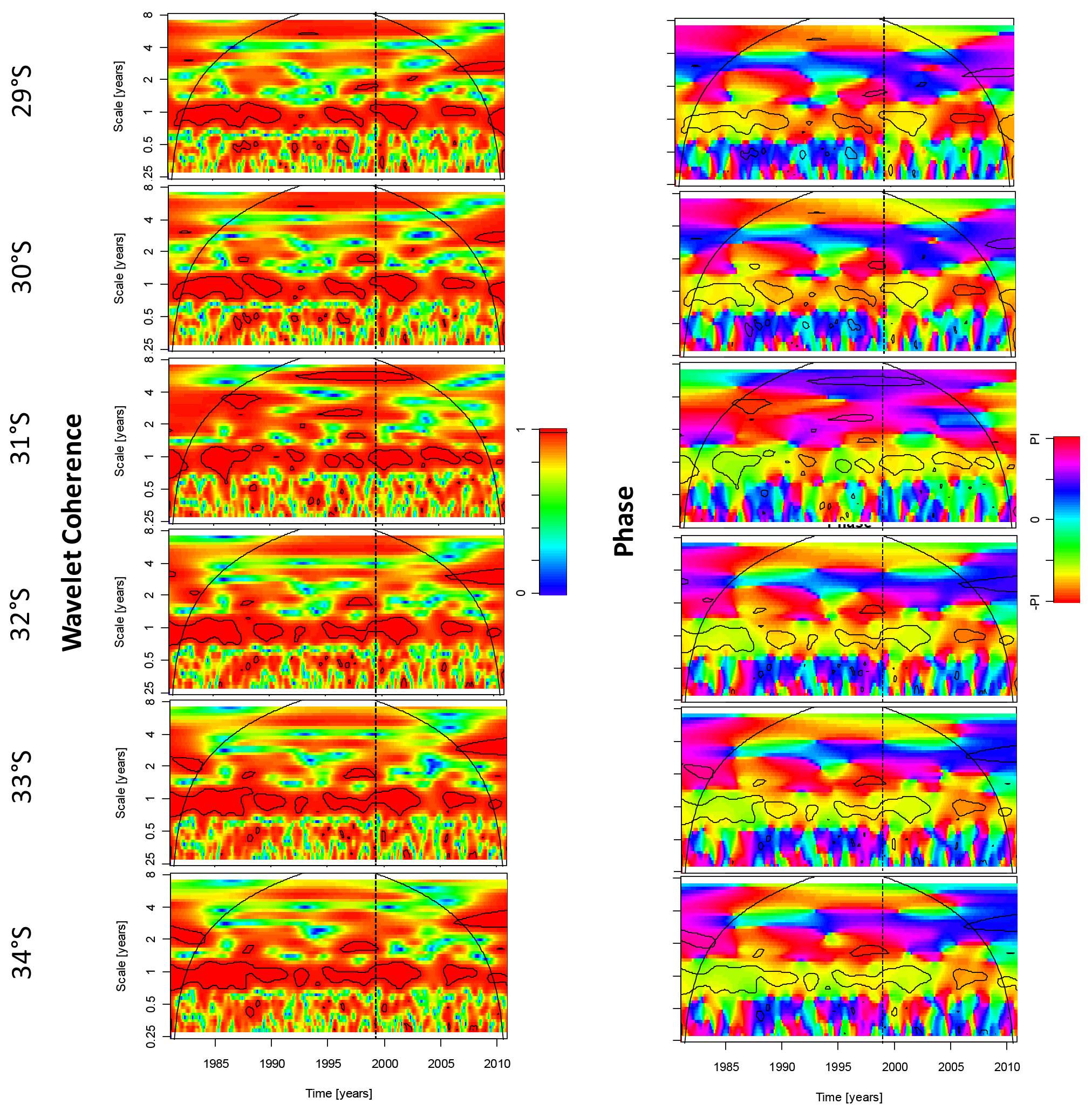

Supplement: Figure S2 — Wavelet coherence and phase between the upwelling index and sea surface temperatures-Niño, computed with data from 1981 to 2010 (included our study time span: 1999–2010, dotted line). The area marked by the black lines indicates the cone of influence where edge effects become important. The solid black contour encloses regions of >95% confidence. (TIF) [file pone.0090276.s003.tif]
